# Modulation of Redox and Immune Responses Following Eight Weeks of Supplementation with a Yeast Cell-Derived Formulation Containing β-Glucans and Micronutrients in Healthy Men

**DOI:** 10.3390/nu18101547

**Published:** 2026-05-13

**Authors:** Daniel König, Markus Gassner, Laura Bragagna, Karl-Heinz Wagner, Aloys Berg

**Affiliations:** 1 Faculty of Life Sciences, Department for Nutrition, Section for Nutrition, Exercise and Health, University of Vienna, 1150 Vienna, Austria; daniel.koenig@univie.ac.at (D.K.); markus.gassner@univie.ac.at (M.G.); 2 Vienna Doctoral School of Pharmaceutical, Nutritional and Sport Sciences, University of Vienna, 1090 Vienna, Austria; laura.bragagna@univie.ac.at; 3 Centre for Sports Science and University Sports, Department of Sports Science, Section for Nutrition, Exercise and Health, University of Vienna, 1150 Vienna, Austria; 4 Department of Nutritional Sciences, University of Vienna, 1090 Vienna, Austria; karl-heinz.wagner@univie.ac.at; 5 Faculty of Medicine, University of Freiburg, 79117 Freiburg, Germany

**Keywords:** yeast cell-derived supplement, β-glucans, exercise stress, inflammatory regulation, infection susceptibility

## Abstract

**Background/Objectives**: Nutritional strategies targeting redox and immune pathways may help to stabilize redox hemodynamics and support immune competence. Controlled physiological stress models allow examination of how nutrients influence dynamic antioxidant and inflammatory responses. **Methods**: In this randomized, double-blind, placebo-controlled trial (RCT), 39 healthy, moderately active men (supplement group: *n* = 20; placebo group: *n* = 19) received a yeast cell-derived formulation containing β-glucans and micronutrients or placebo for 8 weeks. Two standardized high-intensity interval training (HIIT) sessions (PRE/POST) transiently induced oxidative and inflammatory stress. Outcomes included reactive oxygen species (ROS; whole-blood EPR), total antioxidant capacity (FRAP), superoxide dismutase (SOD), interleukin-6 (IL-6), tumor necrosis factor-α (TNF-α), and upper respiratory tract infection (URTI) incidence and duration. **Results**: Prior to the intervention period, acute supplement intake resulted in a more pronounced reduction in ROS from 0′ to 60′ compared with placebo (−6.2%; *p* ≈ 0.14). After eight weeks, fasting FRAP increased only in the supplemented group (*p* < 0.01). Mixed-model repeated-measures ANOVA demonstrated significant time × group interactions for FRAP in both PRE and POST assessments, indicating differential temporal trajectories. The chronic FRAP increase correlated with the acute ROS reduction (*p* < 0.05; r^2^ = 0.21). SOD activity was higher in the supplemented group at 60′ in the POST assessment (*p* < 0.05), and a significant time × group interaction was observed for SOD in POST. TNF-α decreased across the intervention in participants with elevated baseline values, whereas individuals with low initial concentrations showed no change. The supplemented group reported shorter URTI duration (−1.4 days; d = 0.34) and fewer prolonged episodes (>10 days: 5% vs. 15.8%), although these differences were not statistically significant. **Conclusions**: Eight weeks of supplementation with a yeast cell-derived formulation containing β-glucans and micronutrients was associated with differences in selected redox-related markers, including FRAP and SOD, without altering exercise-induced ROS dynamics. The observed patterns suggest subtle modifications in antioxidant-related response characteristics under standardized physiological stress. These findings warrant further investigation in larger and more heterogeneous cohorts, particularly in populations exposed to higher oxidative or inflammatory burden.

## 1. Introduction

Reactive oxygen species (ROS) are continuously produced under physiological conditions and rise markedly during metabolic overload or inflammation, reflecting their dual role as signaling mediators and potential sources of cellular damage. At physiological levels, ROS contribute to mitochondrial biogenesis, immune function, angiogenesis, and cellular adaptation [1,2,3], whereas excessive or insufficiently neutralized ROS promote oxidative damage, chronic inflammation, and various metabolic and neurodegenerative disorders [4,5,6]. Thus, the biological impact of ROS depends on the balance between oxidant production and antioxidant defense, and disturbances in this equilibrium may impair immune competence and increase susceptibility to infections under stress [7]. High-intensity exercise provides a well-controlled model to examine redox–inflammatory dynamics. Strenuous activity transiently elevates mitochondrial and non-mitochondrial ROS formation alongside metabolic and cytokine responses [8]. While these short-lived perturbations support adaptive signaling, excessive ROS exposure can impair recovery and transiently suppress immune function [9,10].

To maintain redox homeostasis, the human body relies on a hierarchical antioxidant network. Enzymatic defenses—including superoxide dismutases (SOD1–3), catalase, glutathione peroxidases, and peroxiredoxins—constitute the first line of protection by converting reactive intermediates into less harmful species. Non-enzymatic antioxidants such as glutathione, uric acid, bilirubin, ascorbate, tocopherols, and dietary polyphenols act as complementary low-molecular-weight scavengers [11]. The coordination of these systems determines whether ROS act as adaptive messengers or accumulate to damaging levels. Acute exercise challenges both components, and the magnitude and timing of enzymatic (e.g., SOD) and non-enzymatic (e.g., ferric-reducing ability of plasma, FRAP) responses are critical for redox recovery and immune resilience [12].

Our group recently demonstrated—using direct electron paramagnetic resonance (EPR) spectroscopy—that resistance-based high-intensity interval training (HIIT) induces reproducible elevations in ROS and interleukin-6, confirming its suitability as a standardized oxidative challenge for investigating nutritional modulation [8]. EPR allows time-resolved quantification of radical formation and overcomes the limitations of indirect oxidative markers [11,12,13].

Nutritional strategies have long been explored as modulators of redox and immune regulation. Although pharmacological doses of isolated antioxidants may blunt redox-sensitive signaling [14,15], moderate, food-derived or multi-component formulations can enhance antioxidant efficiency while preserving physiological ROS dynamics [16,17]. The supplement investigated here combines fermented enzyme-yeast extracts with yeast-derived β-glucans-branched β-(1,3)/(1,6)-linked polysaccharides recognized by Dectin-1 and Toll-like receptor 2 and known to modulate macrophage activity and cytokine responses [18,19,20]. Additional B-vitamins, trace elements, and polyphenolic constituents may further support enzymatic defenses (SOD, catalase, glutathione peroxidase) and Nrf2 (Nuclear factor erythroid 2-related factor 2)-mediated cytoprotective gene expression.

Despite extensive work on individual antioxidants, human studies integrating direct radical detection with enzymatic and non-enzymatic antioxidant indices remain scarce. By combining EPR spectroscopy with biochemical and inflammatory endpoints, the present study aimed to characterize how an eight-week yeast-derived β-glucan–micronutrient complex modulates systemic redox–immune dynamics under standardized oxidative stress.

Accordingly, this randomized, double-blind, placebo-controlled trial investigated whether supplementation alters ROS kinetics, total antioxidant capacity (FRAP), enzymatic defense activity (SOD), inflammatory mediators (TNFα, IL-6), and the incidence and duration of upper respiratory tract infections (URTIs) in healthy men.

## 2. Methods

### 2.1. Study Design and Participants

**Figure 1:**
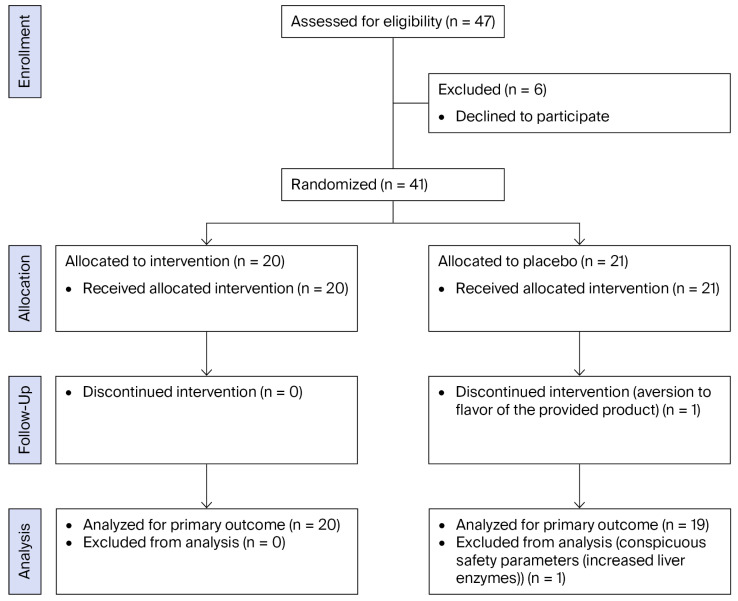
Consort Flow Diagram.

This randomized, double-blind, placebo-controlled, parallel-group trial was conducted over eight weeks to evaluate the effects of supplementation with a yeast cell-derived combination preparation containing β-glucans and micronutrients (Zell Oxygen^®^ Immunkomplex, Dr. Wolz Zell GmbH, Geisenheim, Germany) on redox and immune responses. Forty-seven healthy, moderately active men (less than 3 h of regular physical activity per week), aged 18–40 years, with a BMI between 18.5 and 27.5 kg/m^2^ were screened (Figure 1); thirty-nine completed the study (supplement group: *n* = 20; placebo group: *n* = 19). Participants were non-smokers, free of chronic diseases, and refrained from any nutritional supplements or medication for 12 weeks prior to enrollment. Written informed consent was obtained from all participants. All procedures conformed to the Declaration of Helsinki and were approved by the institutional ethics committee of the University of Vienna (No. 01078, 21 February 2024), in addition the study was registered as a clinical study at the Deutsches Register Klinischer Studien (drks.de; no. DRKS00038407; date: 13 November 2025).

### 2.2. Intervention

Participants received either a yeast cell-derived combination preparation containing β-glucans and micronutrients (30 mL/day) or a taste-matched, ingredient-free placebo for eight weeks. The active supplement combined fermented enzyme yeast providing endogenous redox-active cofactors together with B-vitamins and trace elements, complemented by β-glucans known to prime innate immune signaling and enhance antimicrobial defense pathways [18,19,20]. The full composition of the supplement is shown in Table 1. Compliance, verified through daily intake logs and returned bottle counts, exceeded 95%.

### 2.3. Exercise Model and Blood Sampling

The study was conducted at the University of Vienna (Vienna, Austria). All sessions started at 8:30 a.m. To minimize confounding influences, participants abstained from exercise and alcohol for 48 h and fasted overnight (12 h) before each experimental day. Following arrival, participants remained seated for 2 h under standardized resting conditions, with 0.5 L of water consumed 1 h before and immediately after the first blood sampling to ensure adequate hydration.

Venous blood was drawn at four standardized time points: baseline prior to ingestion (0 min), 1 h post ingestion/pre-exercise (60 min), immediately after the resistance circuit high-intensity interval training (HIIT, 90 min), and after 30 min of recovery (120 min). Participants rested in a seated position between samplings and reclined on an examination table during blood collection.

Each participant completed this entire protocol twice: once before the 8-week supplementation period (“PRE”) and once afterward (“POST”). This PRE–POST design allowed us to (i) characterize acute redox and inflammatory responses to the HIIT challenge under standardized conditions and (ii) determine whether eight weeks of supplementation altered baseline status or the temporal response profile across the four sampling time points.

**Figure 2:**
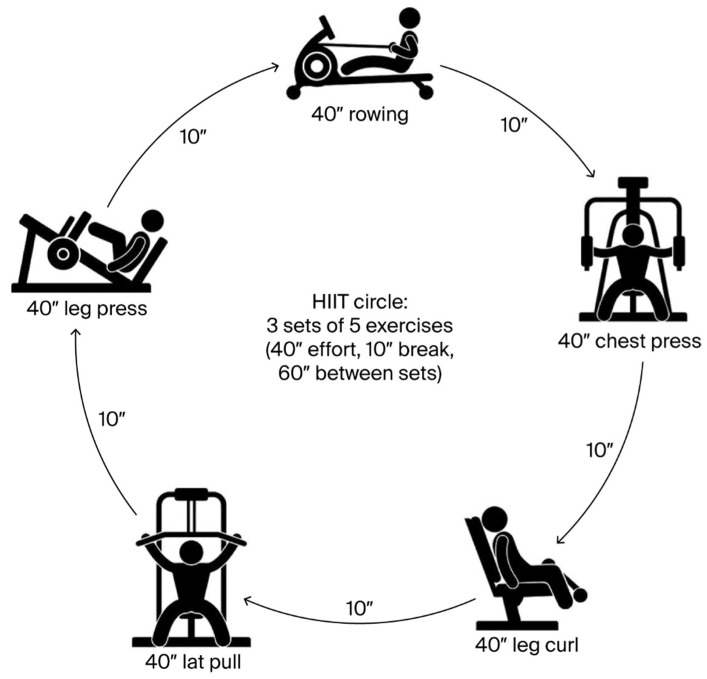
Schematic representation of the high-intensity interval training (HIIT) session performed by participants. ″ = seconds.

Each participant completed two identical resistance-based HIIT sessions—before and after the 8-week intervention—to induce a reproducible oxidative and inflammatory stress response [8]. Each session consisted of a HIIT circle (3 sets of 5 exercises, with an intensity of 50% of individual 1-RM (Figure 2). Prior to the first intervention day, each participant had to perform a 5-RM test, to determine their individual strength for each of the resistance exercises. Consecutively, 1-RM was estimated using the Brzycki equation.

Venous blood was used for direct ROS assessment, while plasma was analyzed for antioxidant and inflammatory markers. To control subjective perception of exercise intensity, the BORG scale was used.

### 2.4. Biochemical and Clinical Endpoints

Primary endpoints comprised reactive oxygen species (ROS) generation measured by electron paramagnetic resonance (EPR) spectroscopy in whole blood, based on a previously described protocol [8,13]. In brief, at each measurement time point, 25 µL of venous blood was collected. A 25 µL volume of Krebs–HEPES buffer containing the CMH spin probe (1-hydroxy-3-methoxycarbonyl-2,2,5,5-tetramethylpyrrolidine, Noxygen, 400 µmol/L) was added. The mixture was vortexed, and 40 µL was immediately transferred into a Hirschmann capillary tube.

Capillaries were placed into the Bruker EPR spectrometer (Bruker, Karlsruhe, Germany), followed by a 60 s incubation period at 37 °C, maintained by a temperature and gas controller (Noxygen, Germany). Within the sample, CMH interacts with both intra- and extracellular ROS, forming the stable CM radical. ROS formation was quantified using the CMH spin probe and expressed as µmol/min, like previous work [13]. EPR measurements were performed using the following settings: center field 3489.510 G, sweep width 60.0 G, static field 3489.527 G, frequency 9.779333 GHz, power 20.97 mW, receiver gain 1.00 × 10^3^, modulation frequency 86.0 kHz, modulation amplitude 2.13 G, modulation phase 1.68, offset 1.00, time constant 40.96 ms, conversion time 10.24 ms, sweep time 5.24 s, resolution (X) 512, resolution (Y) 6, number of X-scans 10.

### 2.5. Ferric Reducing Ability of Plasma (FRAP) Assay

To assess total antioxidative capacity of plasma, the ferric reducing ability of plasma (FRAP) assay was applied based on the method of Benzie and Strain [11]. For quantification of antioxidative capacity, a FeSO_4_·7H_2_O standard series (range 100–2000 µM) was prepared. Trolox, a vitamin E analog, was applied as the positive control. Before analysis, plasma samples were thawed from −80 °C. For the assay, 10 µL of EDTA plasma or 10 µL of FeSO_4_·7H_2_O standards, 30 µL of distilled water, and 300 µL FRAP reagent (25 mL 300 mM acetate buffer, 2.5 mL 10 mM TPTZ solution, 2.5 mL 20 mM FeCl_3_·6H_2_O) were added to each well. After incubation for six minutes at 37 °C, absorbance was measured at 595 nm (CLARIOstar-plus microplate reader, BMG Labtech, Ortenberg, Germany). FRAP results were expressed as mM Trolox equivalents (TEAC).

### 2.6. Interleukin 6

Plasma IL-6 concentrations were determined by using an ELISA according to the manufacturer’s instructions (Human IL-6 DuoSet, Bio-Techne Ireland Limited, Dublin 11 Y1KF, Ireland). All analyses were performed in duplicate, and quality-control procedures followed the laboratory’s internal standards.

### 2.7. Body Composition

Body mass index (BMI) and body fat percentage were measured in the fasted state. Bioelectrical impedance analysis (BIA) was performed in accordance with ESPEN guidelines [21]. Measurements were conducted under standardized conditions (room temperature 20–22 °C) between 8:30 a.m. and 11:30 a.m. Participants were instructed to avoid physical activity and alcohol consumption for 48 h prior to assessment.

### 2.8. TNF-α and SOD

Plasma TNF-α concentrations were quantified using ELISA’s according to the manufacturer’s instructions (Human TNF-alpha DuoSet, Bio-Techne Ireland Limited, Dublin 11 Y1KF, Ireland).

SOD activity was determined spectrophotometrically using a xanthine/xanthine-oxidase-based assay measuring the inhibition of superoxide-driven formazan formation. All analyses were performed in duplicate, and quality-control procedures followed the laboratory’s internal standards.

### 2.9. URTI and Dietary Assessment

The incidence and duration of upper respiratory tract infections (URTIs) were self-reported by participants using standardized log sheets. Dietary intake was assessed using a standardized 24 h dietary recall conducted by trained staff. Portion sizes were estimated with validated photo guides, and nutrient intake was analyzed using the nut.s science software 2026 (nutritional Software GmbH, Vienna, Austria), based on the German Nutrient Database (BLS). Additional outcomes included subjective exertion (Borg scale), heart rate, and standard laboratory safety parameters.

### 2.10. Statistical Analysis

Data were analyzed using SPSS v24 (IBM, Armonk, NY, USA). Normality was verified by the Shapiro–Wilk test. Descriptive data are reported as mean ± standard deviation. To examine the acute responses to the HIIT protocol and possible changes after the 8-week supplementation period, the PRE and POST datasets were analyzed separately. For each biomarker (ROS, FRAP, SOD, IL-6, TNF-α), a mixed repeated-measures ANOVA with 2 (group: Placebo vs. Verum) × 4 (time: 0′, 60′, 90′, 120′) factors was conducted. This design allowed assessment of acute exercise effects over time and potential differences between the two groups within each measurement phase. In addition, PRE→POST change scores (Δ) were calculated for each time point. These Δ values were examined in additional repeated-measures ANOVA including the within-subject factor Δ_time (0′, 60′, 90′, 120′) and the between-subject factor group. Between-group differences were evaluated using independent *t*-tests. Within-group changes were assessed by paired *t*-tests. Effect sizes were calculated as Cohen’s d or Hedges’ g. A 2-sided *p* < 0.05 was considered statistically significant.

## 3. Results

### 3.1. Participant Characteristics

A total of 40 subjects were included and 39 participants completed the intervention (supplement group: *n* = 20, placebo Group: *n* = 19). Baseline characteristics are presented in Table 2.

Baseline anthropometric and physiological parameters were comparable between groups, and no differences were observed in age or training status (hours/week). In addition, no significant differences in baseline energy or macronutrient intake were observed between the two groups. Compliance, verified by returned supplement bottles, exceeded 95%; no adverse events or clinically relevant laboratory abnormalities occurred during the intervention. Hematological, hepatic, and renal parameters remained within reference ranges for all participants throughout the study.

### 3.2. Oxidative, Antioxidant and Inflammatory Markers

At baseline, no significant group differences were present for any oxidative or antioxidant parameter.

### 3.3. Reactive Oxygen Species (ROS)

Immediately following ingestion on Day 1, a more pronounced decline in ROS was observed in the supplement group (Table 3; −6.23%; *p* ≈ 0.14), consistent with an acute antioxidant response. No significant group differences could be observed immediately and 30 min. after the HIIT session.

At both assessment phases (PRE and POST), ROS showed a time effect during and after HIIT (†). ROS increased from 0′ to 90′ and declined toward 120′. This pattern was observed in both groups. No significant group differences or time × group interactions were found in either phase. PRE→POST change scores (Δ) did not differ between groups at any time point.

### 3.4. Ferric Reducing Ability of Plasma (FRAP)

**Figure 3:**
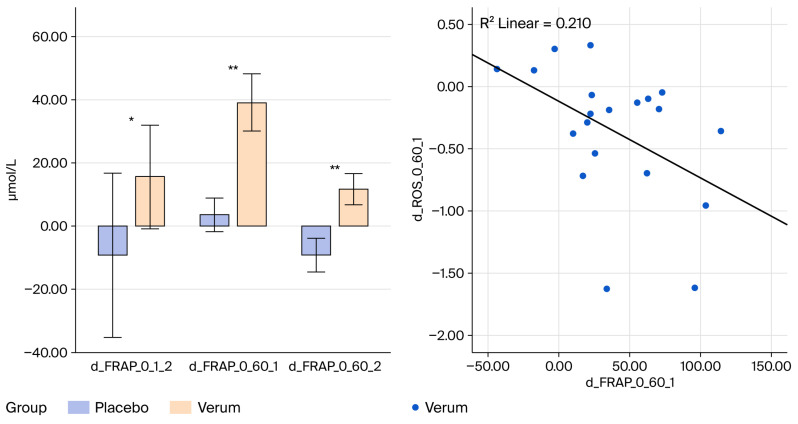
Change in FRAP concentrations at baseline before and after the supplementation period (d_FRAP_0_1_2) as well as after 60 min after intake of the supplement vs. placebo, both before (d_FRAP_0_60_1) and after the supplementation period (d_FRAP_0_60_2). Correlation between the increase in FRAP following supplement intake (d_FRAP_0_60_1) and the decrease in ROS during that period (d_ROS_0_60_1). Across all participants, the magnitude of ROS reduction correlated positively with the increase in FRAP (*p* < 0.05; r^2^ = 0.21), indicating a functional coupling between reactive species quenching and enhanced reducing capacity. * *p* < 0.05; ** *p* < 0.01.

FRAP increased across the exercise protocol (Table 4; †) in both phases, with the highest values observed at 120′. The mixed-model repeated-measures ANOVA showed a significant time × group interaction (*) in both the PRE (*p* < 0.05) and POST assessments (*p* = 0.013), indicating that the temporal response profiles differed between groups before and after the 8-week supplementation period. In both phases, the main effect of time was significant, whereas no main effect of group was detected. The PRE→POST change-score analyses (Δ) showed no significant differences between groups at any time point. Independent-samples comparisons provided additional descriptive information. Only the Verum group showed a significant 0′→60′ increase in FRAP on the respective test days during both the PRE and POST assessments, whereas no significant early change was observed in the Placebo group. Furthermore, baseline FRAP values at 0′ were significantly higher in the Verum group after 8 weeks compared with the Placebo group (Figure 3). These findings reflect higher absolute FRAP concentrations in the Verum group at both baseline and during the early time course, although these absolute differences were not mirrored by group main effects in the RM-ANOVA models.

### 3.5. Superoxide Dismutase (SOD)

SOD increased over time during the HIIT protocol in both phases, as reflected by significant time effects (Table 5; †) in both the PRE and POST assessments (*p* < 0.001). Before the 8-week supplementation period, no time × group interaction was observed (*p* = 0.294), indicating comparable temporal response patterns between groups. After 8 weeks, however, a significant time × group interaction (*) emerged (*p* = 0.024), showing that the temporal SOD trajectories differed between the Verum and Placebo groups in the POST assessment. No significant group main effects were detected, and PRE→POST change scores (Δ) did not differ between groups.

**Figure 4:**
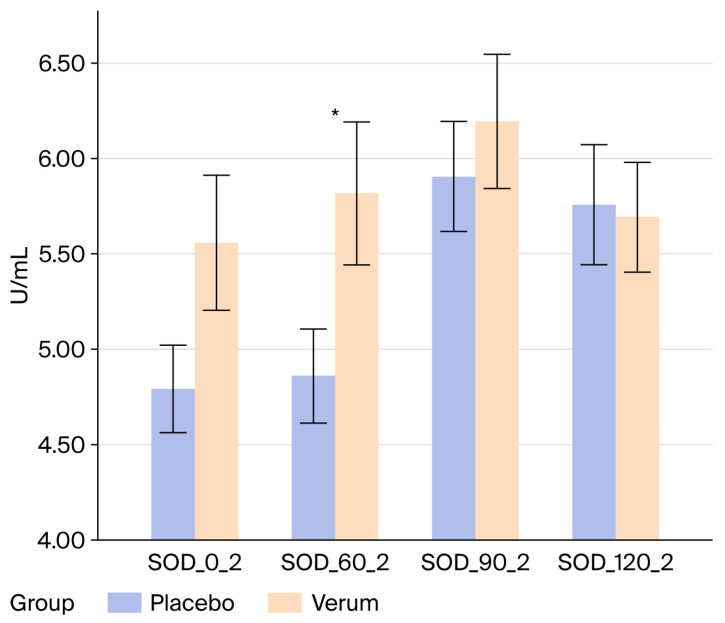
Changes in SOD-concentrations after the 8 weeks supplementation period before (SOD_0_2) and 1 h after the intake of the supplement/placebo (SOD_60_2) and immediately (SOD_90_2) and 30 min after the HIIT (SOD_120_2). * *p* < 0.05.

Additional independent-samples comparisons (Figure 4) showed that baseline SOD concentrations (0′) were higher in the Verum group after the 8-week supplementation period, and that the difference in SOD values at 60′ reached significance in the Verum group compared with Placebo on the POST test day. These differences were not present in the PRE assessment.

### 3.6. Interleukin-6 (IL-6)

IL-6 concentrations showed a gradual increase over the four time points at PRE and POST (Table 6). Main effects of time were significant in both assessments. Group effects and time × group interactions were not detected. Δ analyses confirmed the absence of group-related differences.

### 3.7. Tumor Necrosis Factor-α (TNF-α)

TNF-α showed a modest rise to 90′, followed by a decline at 120′ (Table 7). This temporal pattern was significant in both phases. No significant differences between groups or time × group interactions were found. PRE→POST Δ analyses yielded no group effects.

Across the 8-week supplementation period, TNF-α concentrations decreased in the Verum group, while values in the Placebo group showed no comparable change. Although this reduction did not reach statistical significance in the PRE→POST Δ analyses, the descriptive pattern was consistent. Additional inspection of individual trajectories indicated that this decrease occurred exclusively in participants who presented with elevated TNF-α concentrations at baseline, whereas individuals with low initial values showed no meaningful change.

### 3.8. URTI Incidence and Duration

Over the eight-week period, the supplement group reported fewer upper respiratory tract infection (URTI) days compared with placebo (mean difference = 1.42 days; one-sided *p* = 0.15; Cohen’s d = 0.34). Prolonged URTI episodes lasting more than ten days were approximately threefold less frequent in the supplement group (5% vs. 15.8%). Although these differences did not reach statistical significance, the direction of effects is reported descriptively and requires confirmation in adequately powered studies using objective clinical endpoints.

## 4. Discussion

This randomized, double-blind, placebo-controlled study investigated whether eight weeks of supplementation with a yeast cell-derived combination preparation containing β-glucans and micronutrients modulated markers of redox and immune regulation in healthy, moderately active men. Using a standardized high-intensity interval exercise model to induce transient oxidative and inflammatory stress, three principal findings emerged: (i) FRAP increased significantly in the supplemented group at several baseline and early post-ingestion time points, (ii) SOD activity was higher after the intervention, particularly at baseline and 60′ in the supplemented group, and (iii) changes in FRAP correlated with reductions in ROS. Acute exercise-induced ROS elevations remained detectable, confirming the robustness of the physiological challenge. Overall, these results reflect subtle differences in antioxidant-related response characteristics during oxidative perturbation, while exercise-induced ROS dynamics remained unchanged between groups.

The concurrent increases in SOD activity (mediating superoxide dismutation to hydrogen peroxide) and FRAP suggest improved integration of enzymatic antioxidant defenses and low-molecular-weight antioxidant systems. The positive association between changes in FRAP and ROS reduction further supports a more efficient redox coupling, in which higher extracellular reducing capacity is linked with lower radical accumulation under standardized stress. Collectively, the data indicate subtle differences in antioxidant response characteristics under standardized oxidative stress conditions, without evidence of altered radical generation. These observations are consistent with the concept of redox network adaptability, implying coordinated contributions of enzymatic and non-enzymatic systems to the maintenance of thiol homeostasis and redox signaling stability [1,2,11,22,23]. All effects remained within physiological limits, indicating a modulation rather than a suppression of oxidative dynamics. As total antioxidant capacity assessed by FRAP can be influenced by exercise-induced changes in uric acid, particularly in the post-exercise state, this marker should be interpreted as an integrative measure of extracellular reducing capacity rather than as a specific antioxidant effect. Given that the exercise protocol and sampling schedule were identical in both groups, any acute uric acid-related shifts would be expected to occur comparably, and therefore do not fully account for the observed differences in temporal FRAP response patterns.

The immune and inflammatory results complement this biochemical profile. Mean cytokine concentrations remained within normal ranges, but a decline in TNFα was apparent among participants with elevated baseline levels, indicating inter-individual variability in redox–inflammatory sensitivity. Importantly, this reduction occurred exclusively in individuals presenting with higher pre-intervention TNFα concentrations, whereas participants with low initial values showed no meaningful change—consistent with a modulatory rather than suppressive effect. Such patterns may also be viewed in the context of known mechanistic actions of yeast β-glucans, which can modulate macrophage activity and cytokine coordination through Dectin-1 and related pathways [19,20]. Given that TNFα transcription is regulated by redox-sensitive transcription factors such as NF-κB and AP-1 [7,9,24], this finding is consistent with a systemic redox–immune coupling rather than a purely anti-inflammatory action. Such variability supports the notion that pre-existing oxidative and inflammatory tone determines responsiveness to nutritional or environmental modulation. Exploratory clinical data revealed a tendency toward fewer and shorter URTIs in the supplemented group, although without statistical significance. These observations should not be interpreted as evidence of immune efficacy or clinical benefit, but rather as descriptive findings reflecting baseline-dependent variability in immune-related markers. 

Within these clearly defined limitations, the observed patterns are compatible with an association between redox-related markers and immune-related readouts under standardized physiological stress—an observation warranting confirmation in adequately powered and stratified cohorts using objective endpoints. Together, these data are compatible with the concept of a physiological link between oxidative regulation, inflammatory signaling, and host defense.

A distinctive methodological feature of this work is the use of EPR spectroscopy for direct quantification of short-lived radicals in whole blood. Beyond its analytical precision, EPR allows time-resolved assessment of radical formation under near-physiological conditions, capturing transient changes that conventional end-point markers such as malondialdehyde or F_2_-isoprostanes cannot reveal [11,12,13]. When combined with FRAP and enzymatic defense activity (SOD), this approach enables a complementary characterization of redox-related markers under standardized stress conditions. The standardized high-intensity interval protocol further enhanced internal validity by eliciting reproducible oxidative and inflammatory responses in a controlled, time-anchored design [8].

The present findings align with earlier reports describing moderate improvements in antioxidant indices after complex nutrient-based interventions in healthy or active cohorts [16,17,20,25,26,27]. Such approaches are increasingly used to capture integrated physiological responses at the systems level, albeit at the expense of ingredient-specific mechanistic resolution. The integration of direct radical detection with complementary enzymatic and non-enzymatic measures extends this literature by enabling functional interpretation of redox physiology rather than isolated biomarker shifts. The results suggest that changes in redox-related markers reflect coordinated adjustments among multiple antioxidant systems rather than isolated single-parameter effects [3,4,10].

This multidimensional approach offers a basis for characterizing individual patterns of redox efficiency, which may be particularly relevant in populations exposed to recurrent metabolic, oxidative, or inflammatory stress. Future studies should incorporate higher temporal resolution, larger and more heterogeneous cohorts, and multi-omics strategies—including redox proteomics, metabolomics, and transcriptomics—to delineate pathway-specific adaptations. Stratification by baseline oxidative and inflammatory status will likely be essential for understanding inter-individual variability and for identifying subgroups that may benefit most from targeted nutritional or lifestyle modulations. In this context, the present study should be regarded as an exploratory, hypothesis-generating investigation aimed at characterizing redox–immune response patterns under standardized physiological stress.

Although the present trial provides internally consistent evidence across complementary redox markers, several limitations must be acknowledged. The modest sample size limits statistical power for inflammatory and clinical outcomes, and the homogeneous cohort of healthy young men restricts generalizability to women, older adults, or individuals with elevated oxidative or inflammatory burden. Given the number of statistical tests performed, the possibility of type I error cannot be excluded, particularly for secondary and exploratory analyses, and findings should be interpreted within this statistical context. Furthermore, URTI incidence and duration were self-reported, introducing susceptibility to recall bias and underscoring the need for objective verification using standardized diagnostic criteria in future trials. Finally, as with all nutritional intervention studies, the findings reflect the effects of a complex multi-component formulation, precluding attribution to any single ingredient.

Despite these limitations, the methodological precision achieved through direct EPR-based radical quantification, a standardized and reproducible physiological stressor, and the alignment of enzymatic and non-enzymatic antioxidant responses provides a platform for hypothesis generation. By capturing radical kinetics alongside systemic antioxidant responses within a single experimental model, this study provides a structured approach for examining how nutritional factors may modulate redox–immune interactions, potentially contributing to the understanding of inter-individual differences in stress-related redox and immune responses.

## Figures and Tables

**Table 1:** Composition of the Multi-Ingredient Supplement.

Ingredient	Amount per Daily Dose (30 mL)	% NRV	Notes
Beta-glucans from yeast (Saccharomyces cerevisiae)	900 mg	-	
Lycopene	9.3 mg	-	
Enzyme yeast cells Dr. Wolz^®^	16 g	-	≈150 billion cells
Vitamin B1 (Thiamine)	2.2 mg	200%	
Vitamin B2 (Riboflavin)	2.8 mg	200%	
Vitamin B6 (Pyridoxine)	2.8 mg	200%	
Vitamin B12 (Cobalamin)	5 µg	200%	
Niacin (NE)	16 mg	100%	Niacin equivalents
Pantothenic acid	6 mg	100%	
Biotin	100 µg	200%	
Vitamin D3	5 µg (200 IU)	100%	
Vitamin E (as α-TE)	24 mg	200%	α-tocopherol equivalents
Zinc	10 mg	100%	
Selenium	55 µg	100%	

NRV: Nutrient Reference Value.

**Table 2:** Anthropometric variables at baseline.

Variable	Control Group (Mean ± SD)	Verum Group (Mean ± SD)	Significance Between Groups (Baseline)
Age (y)	27.11 ± 3.38	26.90 ± 3.88	n.s.
BMI (kg/m^2^)	23.86 ± 1.85	24.35 ± 1.66	n.s.
Body fat (%)	17.93 ± 5.31	18.62 ± 5.86	n.s.

n.s.—not significant.

**Table 3:** ROS (A.U.; Mean ± SD). Symbols explanations: (†) time-related response during and after stress test; (Δ) pre-post chance-score analysis between groups; (−) no significant differences.

Time	Placebo PRE	Verum PRE	Placebo POST	Verum POST	ΔPlacebo	ΔVerum	Significance
0′	1.947 ± 0.263	2.179 ± 0.443	2.354 ± 0.579	2.371 ± 0.586	+0.407	+0.193	† (PRE), † (POST), − (Δ)
60′	1.775 ± 0.177	1.817 ± 0.309	1.969 ± 0.450	1.926 ± 0.294	+0.194	+0.110	† (PRE), † (POST), − (Δ)
90′	2.542 ± 0.333	2.582 ± 0.406	2.579 ± 0.365	2.644 ± 0.376	+0.037	+0.062	† (PRE), † (POST), − (Δ)
120′	2.012 ± 0.247	1.976 ± 0.189	1.932 ± 0.164	2.069 ± 0.307	−0.079	+0.094	† (PRE), † (POST), − (Δ)

**Table 4:** FRAP (µmol/L; Mean ± SD). Symbols explanations: (†) time-related response during and after stress test; (*) significant time × group interaction; (Δ) pre-post chance-score analysis between groups; (−) no significant differences.

Time	Placebo PRE	Verum PRE	Placebo POST	Verum POST	ΔPlacebo	ΔVerum	Significance
0′	954.44 ± 118.10	956.01 ± 121.64	945.22 ± 135.54	971.64 ± 108.71	−9.22	+15.63	† * (PRE), † * (POST), − (Δ)
60′	957.99 ± 121.95	995.15 ± 121.49	936.04 ± 138.29	983.32 ± 114.06	−21.96	−11.83	† * (PRE), † * (POST), − (Δ)
90′	959.21 ± 128.63	974.41 ± 117.99	933.13 ± 135.00	958.81 ± 121.64	−26.08	−15.60	† * (PRE), † * (POST), − (Δ)
120′	1197.12 ± 131.17	1198.27 ± 146.66	1218.18 ± 152.32	1206.53 ± 175.16	+21.06	+8.26	† * (PRE), † * (POST), − (Δ)

**Table 5:** SOD (U/mL; Mean ± SD). Symbols explanations: (†) time-related response during and after stress test; (*) significant time × group interaction; (Δ) pre-post chance-score analysis between groups; (−) no significant differences.

Time	Placebo PRE	Verum PRE	Placebo POST	Verum POST	ΔPlacebo	ΔVerum	Significance
0′	4.842 ± 0.935	5.064 ± 1.125	4.793 ± 0.992	5.556 ± 1.599	−0.049	+0.492	† (PRE), † * (POST), − (Δ)
60′	4.909 ± 0.858	5.209 ± 0.841	4.861 ± 1.075	5.815 ± 1.683	−0.048	+0.607	† (PRE), † * (POST), − (Δ)
90′	5.718 ± 1.034	6.105 ± 1.221	5.906 ± 1.260	6.195 ± 1.572	+0.188	+0.091	† (PRE), † * (POST), − (Δ)
120′	5.407 ± 1.239	5.370 ± 0.880	5.758 ± 1.372	5.693 ± 1.293	+0.351	+0.323	† (PRE), † * (POST), − (Δ)

**Table 6:** IL-6 (pg/mL; Mean ± SD). Symbols explanations: (†) time-related response during and after stress test; (Δ) pre-post chance-score analysis between groups; (−) no significant differences.

Time	Placebo PRE	Verum PRE	Placebo POST	Verum POST	ΔPlacebo	ΔVerum	Significance
0′	4.11 ± 6.11	5.75 ± 7.88	8.06 ± 9.08	6.63 ± 7.69	+3.95	+0.88	† (PRE), † (POST), − (Δ)
60′	4.68 ± 6.57	5.54 ± 7.66	6.93 ± 8.43	6.26 ± 7.53	+2.24	+0.72	† (PRE), † (POST), − (Δ)
90′	4.96 ± 6.93	6.05 ± 7.61	8.10 ± 8.26	6.97 ± 7.28	+3.14	+0.92	† (PRE), † (POST), − (Δ)
120′	4.79 ± 6.30	6.30 ± 8.06	8.56 ± 9.07	7.11 ± 7.65	+3.77	+0.81	† (PRE), † (POST), − (Δ)

**Table 7:** TNF-α (pg/mL; Mean ± SD). Symbols explanations: (†) time-related response during and after stress test; (Δ) pre-post chance-score analysis between groups; (−) no significant differences.

Time	Placebo PRE	Verum PRE	Placebo POST	Verum POST	ΔPlacebo	ΔVerum	Significance
0′	0.74 ± 2.75	1.80 ± 4.01	0.74 ± 2.08	0.80 ± 2.14	0.00	−1.00	† (PRE), † (POST), − (Δ)
60′	0.95 ± 2.78	2.03 ± 3.80	0.79 ± 1.65	1.20 ± 2.63	−0.17	−0.83	† (PRE), † (POST), − (Δ)
90′	1.61 ± 2.82	2.61 ± 4.99	1.02 ± 1.82	1.80 ± 3.56	−0.59	−0.82	† (PRE), † (POST), − (Δ)
120′	1.06 ± 3.03	1.93 ± 3.97	0.84 ± 1.76	1.05 ± 2.06	−0.22	−0.88	† (PRE), † (POST), − (Δ)

## Data Availability

The original data presented in the study are available on request from the corresponding author due to restrictions (e.g., privacy, legal or ethical reasons).
